# Waiting management at the emergency department – a grounded theory study

**DOI:** 10.1186/1472-6963-13-95

**Published:** 2013-03-12

**Authors:** Lena Burström, Bengt Starrin, Marie-Louise Engström, Hans Thulesius

**Affiliations:** 1 Centre for Clinical Research, Uppsala University, Västmanland County Hospital, Västerås, Sweden; 2 Karlstad University, Karlstad, Sweden; 3 Department of Clinical Sciences Malmö, Division of Family medicine, Lund University, R&D, Kronoberg County Council, Växjö, Sweden

**Keywords:** Waiting, Management, Emergency department, Grounded theory, Focus group, Participant observation

## Abstract

**Background:**

An emergency department (ED) should offer timely care for acutely ill or injured persons that require the attention of specialized nurses and physicians. This study was aimed at exploring what is actually going on at an ED.

**Methods:**

Qualitative data was collected 2009 to 2011 at one Swedish ED (ED1) with 53.000 yearly visits serving a population of 251.000. Constant comparative analysis according to classic grounded theory was applied to both focus group interviews with ED1 staff, participant observation data, and literature data. Quantitative data from ED1 and two other Swedish EDs were later analyzed and compared with the qualitative data.

**Results:**

The main driver of the ED staff in this study was to reduce non-acceptable waiting. Signs of non-acceptable waiting are physical densification, contact seeking, and the emergence of critical situations. The staff reacts with frustration, shame, and eventually resignation when they cannot reduce non-acceptable waiting. Waiting management resolves the problems and is done either by reducing actual waiting time by increasing throughput of patient flow through structure pushing and shuffling around patients, or by changing the experience of waiting by calming patients and feinting maneuvers to cover up.

**Conclusion:**

To manage non-acceptable waiting is a driving force behind much of the staff behavior at an ED. Waiting management is done either by increasing throughput of patient flow or by changing the waiting experience.

## Background

An emergency department (ED) should offer timely care for acutely ill or injured persons that require the attention of specialized nurses and physicians. A patient who arrives at the ED is normally cared for and screened in an urgency triage [1]. A nurse then makes a first assessment of how fast the patient needs to be examined by a physician [2]. Alternatively, a physician makes the first assessment. When many people seek care at the ED waiting can be long. A recent Swedish study showed that 38% of ED patients spend more than 4 hours at the ED with the oldest age group waiting the most [3]. Many jurisdictions have wait time reduction strategies [4], and some have goals to maximize waiting times at EDs to 4–6 hours [5]. Patients have to be cared for fast to ensure diagnosis and treatment. Otherwise, patient safety could be compromised. Apart from safety aspects the total patient experience is important and waiting has potential negative consequences for patients. Either their medical condition may deteriorate, or they could get anxious and worried [6,7]. Patients that spend long time at EDs thus risk experiencing discomfort and a lack of care. The situation is especially difficult for elderly patients arriving alone who cannot speak for themselves [8]. Explanatory information of waiting time duration and the caring attitude of the staff is important for patient satisfaction [9,10]. If there is scant information of how the ED works it is difficult for waiting patients to accept that other patients are prioritized before them. It is also difficult to understand why nurses at times seem idle without apparent tasks [11]. Waiting becomes a problem when patients feel that nothing happens at the ED [12]. Frustration and eventually anger then emerges among patients - a difficult task for the staff to deal with [13,14]. The aim of this study was to explore what is going on at an ED using grounded theory, which epistemologically is in “the context of discovery” as opposed to “the context of justification” [15].

## Method

Classic grounded theory (CGT) is a general research method where both qualitative and quantitative data can be used since ”all is data” [16]. In CGT the task is to give explanatory conceptual names to patterns of human behavior [16-18]. These conceptual names emerge from data through a process of coding, comparing and memoing, and eventually become parts of hypotheses in a theory induced from empirical data. The resulting CGT is a set of probability statements aimed at explaining the behavior that accounts for resolving a main concern for the participants [19]. In CGT behavior not people is categorized [20]. In this study, we wanted to figure out what is going on at the Emergency Department - a basic CGT research question. We used CGT for data collection with the exception that interviews were taped and transcribed, not recommended in CGT. The data collection ended when saturation was reached, i.e. the most recent interviews and field notes did not make any substantial contribution to the conceptual model generated from earlier data [17].

### Participants, data collection, and analysis

Quantitative and qualitative data was collected between 2009 to 2011 at a central hospital ED in Sweden with 53.000 yearly visits and a catchment population of 251.000, called ED1. Mainly quantitative data from two other EDs at hospitals with 91.000 yearly visits and a catchment population of 600.000, called ED2 and ED3 with 65.000 yearly visits and a catchment population of 430.000 was also collected.

**Figure 1:**
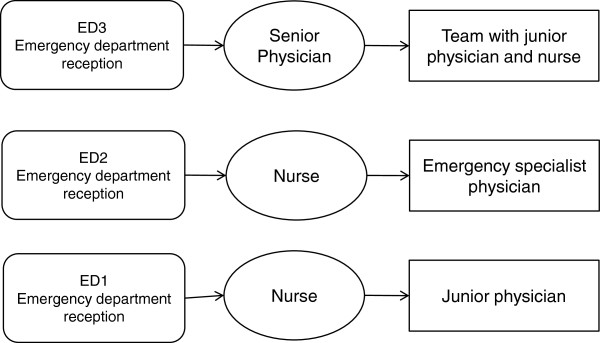
The principal organisation of the three emergency department (ED1, 2, 3) triage models studied.

A patient visiting ED1 takes a queue ticket and a seat in the waiting room. Thereafter a nurse assesses the patient’s health status and makes a triage, i.e. identifies the patients’ level of urgency. The patient then returns to the waiting room and awaits a move either to a stand-by waiting mode in the corridor or to an investigating room. At ED2 a nurse does the first triage with a specialist emergency physician doing the second patient assessment. At ED3 a senior physician does the first triage with a junior physician and a nurse doing a second assessment with a detailed standardized protocol (Figure 1).

The first author (LB) conducted 76 hours of participant observation at ED1 and wrote extensive field notes based on observations that took place both before and after focus group interviews and covered both daytime and nighttime shifts. Also, informal participant observation data and interpreted quantitative data from ED2 and ED3, as reported elsewhere [3], was used in the constant comparative analysis of the three EDs presented in the Discussion. Six focus group interviews were done at ED1, one each with registered nurses, auxiliary nurses, nurse supervisors, junior physicians (locums), specialist physician trainees (registrars), and one with specialist physicians (consultants). Altogether 8 women nurses, 3 men nurses, 3 women physicians, 7 men physicians and 5 women nurse supervisors participated. All except 2 junior physicians had more than 10 years of emergency care and/or other hospital care working experience. The interviews began with “What is happening during a normal day (or night) at the emergency department?”. All data from the six focus group interviews lasted between 90 and 110 minutes and were coded and analyzed according to CGT procedures. We compared the interview data with the observational data as well as the quantitative data consistent with the CGT concept “all is data”. While interviewing we got ideas of what to ask next and generated more specific questions for subsequent interviews. This is called theoretical sampling - “the process of data collection for generating theory whereby the analyst jointly collects, codes, and analyses his data and decides what data to collect next and where to find them, in order to develop his theory as it emerges” [16,17]. We wrote field notes after each interview and analyzed the transcribed interviews and the field notes line by line separately from each other. We began coding the data in every way possible and asked a set of questions to our data: “What are these data a study of?”, “What category does this incident indicate?”, “What is actually happening in the data?”, “What is the main concern of the respondents and what accounts for how this concern is continually resolved”? The purpose of these questions was to keep the analyst theoretically sensitive and transcending when analyzing, collecting, and coding data [16]. The codes were the basic source for concept generation. Thus we generated concepts representing underlying patterns in the data. We compared concepts to other concepts and new incidents in the data, and “Waiting Management” eventually emerged as the core pattern of behavior resolving the main concern, i.e. the core variable or core concept. Hereafter, selective coding was done to mark off the coding to variables related only to the core concept. The core concept was thus a template for further data collection and theoretical sampling [16,17]. We wrote theoretical memos, in the shapes of text and figures, during the whole analytic process. Memos are the “theorizing write-up of ideas about substantive codes and their theoretically coded relationships as they emerge during coding, collecting and analyzing data and during memoing” [16]. During the sorting of the memos, we sought relationships between categories and the core category [21]. We then wrote up the memos to a theory as a last stage of the grounded theory methodology [16,17]. According to CGT principles, we did a literature review after the substantive theory was formulated, using the literature as another source of data integrated into the constant comparative process [16].

### Ethics

Verbal and written information regarding the aim and procedure was given to all participants who were informed that they were free to withdraw from the study at any time and without declaring any reason to do so. The ED staff was informed orally about the observational study before it started and we obtained oral informed consent from staff that was interviewed. The Research Ethics Committee at Uppsala University, Sweden, approved the study for all three EDs (Approval number: 2009/414).

## Results

The main concern at the ED is to reduce the patients waiting time. It is done either by increasing throughput of patient flow by structure pushing and shuffling around patients, or by changing the experience of waiting by calming patients and feinting to cover up. The staff makes a distinction between acceptable and non-acceptable waiting time. Signs of non-acceptable waiting are physical densification, contact seeking, and the emergence of critical situations. The staff reacts with frustration, shame, and eventually resignation when they cannot reduce non-acceptable waiting.

Waiting at ED1 takes place in the waiting room, in the stand-by waiting mode and in the treatment rooms. Standby waiting occurs after the patient has been examined by a nurse and is waiting to get into a treatment room. It can take place in the main waiting room or in the corridors of the ED1 and is intended for patient assessments when no treatment rooms are available in order to increase the patient flow and safety. Depending on the severity of their condition patients who arrive by ambulance enter a trauma room, a treatment room, or the stand-by waiting mode. The patient first meets a physician in a room where investigations, tests, and treatments are done and where the patient waits for test results.

“Emergency department, please wait” is a common response when you phone an ED. This may sound like a paradox, but waiting is what characterizes EDs in general since it is difficult to assess the influx of patients. Patients who come to the ED require a prompt treatment. And a key concern of the staff therefore is to reduce the waiting time.

### Field notes from the waiting room

Twenty-two adults and three children are in the waiting room at the ED; ten of them appear to be accompanying visitors. Most patients entering the waiting room seem confused and are looking for an information desk. Eventually, they find an information board telling them to take a numbered ticket, sit in the waiting room, and wait for the staff. People with chest pain, excessive bleeding, or respiratory problems, are advised to ring a bell at the reception. Patients who have a referral from a family physician are confused. They think that a referral means that they will get help immediately. From a conversation between a patient and an accompanying person I understand that their waiting has been long. The waiting time for a first assessment is approximately one hour and in the standby mode between two to three hours. People wonder how long they will have to wait before they can see a physician. Nurses are walking back and forth to get an overview of what happens in the waiting room. They want to do a first assessment of the patients as soon as possible. Some people are complaining loudly about the waiting time being too long. One patient is exhausted with pain and is lying down on a couch. A nurse comes to hand out painkillers.

During my hours in the waiting room no-one in the staff provides information about why people are waiting or what they are waiting for. On a TV-monitor sitting on a wall visible only from one angle, scrolling information in Swedish says that the sickest persons are dealt with first. I eventually notice that those with orthopaedic injuries are waiting longer and longer in the standby mode after the initial assessment. The flow appears to have stalled. The frustration of nurses walking in and out of the reception is obvious.

### Acceptable and non-acceptable waiting distinction

Waiting management at the ED starts with a distinction between acceptable and non-acceptable waiting. Acceptable waiting is perceived as justifiable, and with the patient agreeing to wait. Non-acceptable waiting is judged as non-justifiable. The definition of what is a non-acceptable waiting time is determined in an interactive process between staff and patients, where the staff is observant upon different signs from patients and accompanying persons. The staff not only pays attention to changes in the patients’ health status but also signs of worry, and perceptions of their experiences of non-acceptable waiting has developed over time. There are several types of reactions the staff considers when judging if waiting is non-acceptable. One reaction to waiting comes from the patients’ *health worries*. Patients who seek help at the ED want to think that they leave their health in the hands of experts who care for them in a safe and optimal way. If the waiting time from arrival to care is long this increases the patients’ fear of a worsening health. Another reaction comes from expectations of a fast visit or a *quick fix*. Patients are then not worried so much about their health, but are filled with norms and expectations of what constitutes an acceptable waiting and want to see a physician fast. Waiting is then perceived as non-acceptable when nothing happens or happens slowly at the ED. Also, if patients are ignorant about, or do not understand why they are waiting, the situation is considered inefficient.

### Signs of non-acceptable waiting

**Figure 2:**
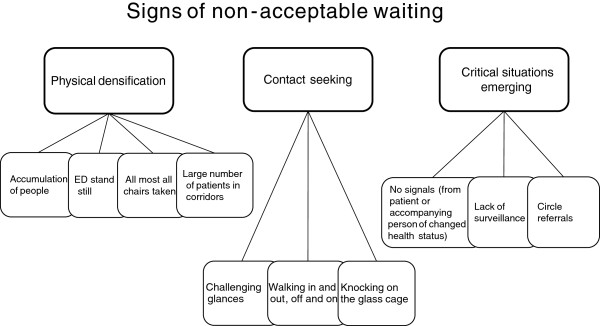
Signs of non-acceptable waiting at an emergency department.

Signs of non-acceptable waiting are physical densification, contact seeking behavior and the emergence of critical situations (Figure 2).

*Physical densification* refers to an *accumulation of people* at the ED making it more difficult to survey patients, and this increases the waiting time. Physical densification is thus the gathering of people when the influx of patients is greater than the outflux, or at peaks when many people seek help at the ED at once. This densification may lead to a stop in the flow of patients and a sense of complete *standstill* at the ED. A standstill results in people remaining in the waiting room or in corridors where more people will be arriving, and soon the ED is overcrowded. One obvious sign of physical densification is that *almost all chairs are taken*. Both the regular and spare seats are filled in the entrance area, sometimes even outside the entrance. Another sign of physical densification is the presence of *large numbers of patients in corridors*. They are all waiting to get into a treatment room. Patients in the corridors and in the treatment rooms remain where they are. The ward staff has not fetched patients who are waiting to move to hospital wards. Reasons for a standstill can be the arrival of seriously injured patients or that a physician is performing a surgery. Or the hospital ward staff is understaffed or too busy to come and collect their admitted patients. There are times when many patients arrive at once as if they came with a bus. This is called "the eleven o'clock bus" or the "four o'clock bus" by the staff.

*Contact seeking* behavior is another sign of non-acceptable waiting characterized by patients or accompanying persons repeatedly trying to connect with the staff to alert them about their existence at the department, and about their state of health. Recurrent attempts to make contact with the staff by appealing or *challenging glances* are examples of contact seeking. Physical activities such as *walking to and fro* without making fuss, or patients going *in and out of the treatment room* are other examples. To tap the *"glass cage"*, i.e. the reception, is also a way to connect even if the signs say that patients are supposed to wait in the waiting room until the staff gives them further notice.

#### Critical situations

People who seek help at the ED are acutely ill with a more or less bad health status. Therefore it is important to be cautious of critical health status changes. *Critical situations* are present if patients are very ill or show health status deterioration.

#### No signaling

Critical situations may arise when a patient or an accompanying person, for different reasons, does not signal a health status change, or there is a lack in the health status surveillance. One reason why patients or accompanying persons do not signal a health status change may be that they do not want to disturb, or that they are waiting in the wrong place. Sometimes patients with serious health conditions who should have been cared for at once are found in the waiting room without having contacted the staff. This situation may arise since patients on a sign in the waiting room are asked to self-evaluate their health status based on written instructions without any support from the staff. According to the instructions chest pain, large bleedings or breathing problems are reasons for patients to alarm the staff by ringing a bell at the reception. Otherwise patients are asked to take a queue number and sit in the waiting room. The patients’ interpretation of the instructions decides if they sit and wait or contact the staff. Language barriers are thus reasons for critical situations to arise.

Another critical situation may arise when patients have extensive care needs but no accompanying person who can alert the staff of a worsened health condition. This mostly applies to older people who arrive without accompanying persons and have difficulties to communicate a worsened health condition. Also, older patients are more inclined to not wanting to disturb and cause trouble, but are patiently waiting for the staff to arrive.

#### Lacking surveillance

A worsened health status may cause a critical situation to emerge in the waiting room when a patient’s health condition is not well surveyed. When patients arrive at the ED, a nurse first examines them to find out the reason for their visit and their health status. Then, patients are prioritized according to how urgently they need to see a physician. If the waiting time is long then the patients’ condition may worsen and there may be a need to repeat the prioritization examination in order to avoid critical situations. A critical situation will eventually occur if there is no staff in the waiting room to watch the patients. The staff would notice if someone tries to contact them, or lacks the ability to contact them.

Patient surveillance is also failing during *physical densification*, which may cause critical situations to occur. It is then difficult to sustain a necessary overview of corridors and treatment rooms. Many people are then moving in a limited space, and the staff has little time to see to patients in treatment rooms before it is time for the physician’s examination.

#### Circle referrals

Another serious risk of critical situations occurring is when referrals are going in circles. At busy hours, and during staff shortage, examining physicians note that patients have many symptoms. Some handled by that physician’s own department, and other symptoms by other departments. If patients then are referred to another department at the ED, this temporarily relieves the own department’s workload. But, the receiving department may then refer the patient to yet another department. Patients in this situation are called “Old maids” (from the card game), since everybody wants to get rid of them. This eventually becomes a critical situation since patients get stuck between different departments and are not safely surveyed.

### Waiting management

**Figure 3:**
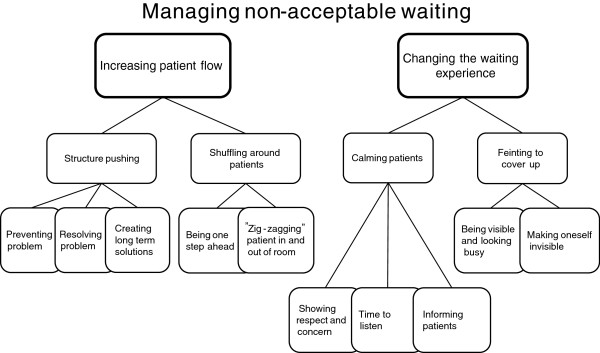
Managing non-acceptable waiting at an emergency department.

Waiting management of non-acceptable waiting is done either by reducing the actual waiting time by *increasing the patient flow,* in order to make the work run smoother or by *changing the waiting experience* (Figure 3).

### Increasing patient flow

When waiting times increase it is necessary to increase the flow of patients and patient turn over. The main reasons for a slow flow are too many patients arriving simultaneously, not enough treatment rooms, not enough experienced physicians, lacking or broken equipment, and not enough hospital beds. The ED staff is either structure pushing or shuffling around to increase the flow throughput and to increase the smoothness of work activities.

#### Structure pushing

This expression used by the staff refers to different strategies to increase patient flow, which in turn will prevent physical densification, contact seeking behavior and the emergence of critical situations, eventually to reduce the risk of jeopardizing patient safety. The staff is structure pushing for three reasons: *to prevent problems, to resolve problems,* and *to create long-term solutions*.

One example of how *to prevent problems* is to avoid flow obstruction and increase patient flow by signaling when both the waiting room and the stand by waiting mode are becoming crowded and the staff is losing overview of the patients. The staff responds by keeping up a fast and smooth flow of patients. To decrease the risk of an obstructed patient flow, the physician acts in a preventive way by ordering extra lab tests. This is done both for their usefulness, and to avoid the risk of having to wait two more hours for future possible tests that would be needed later. By repeatedly calling the blood works lab and the radiology department and pressing them to deliver faster results is another way to try to prevent a work standstill.

*To resolve problems* means that the staff is watching out for emerging problems that need an immediate resolution. This starts when densification has taken place, during on-going recurrent contact seeking behavior and when the risk of critical situations is high. Problem solving work aims at resolving difficult situations here and now. To resolve problems one may sometimes have to push the immediate care structure. Nurses push physicians and tell them to act and reprioritize patients since waiting time has become too long. Physicians are then forced to increase their speed and take care of more patients. The flow has to be efficient with preparedness for the next patient arriving being sicker than the previous one. Another action taken by nurses is when they request another physician with higher competence, or at least any other physician, so that more patients can be examined and cared for. If there are not enough physicians with the right competence to decide what will happen to waiting patients, the ED will soon suffer a standstill.

One common obstacle of the ED flow is a lack of hospital beds. This is a bottleneck when patients are admitted to the wards and calls for further structure pushing. The staff then demands that physicians contact the hospital wards to request them to collect patients that have been admitted. Patients left behind at the ED are occupying space and this impairs the overview.

Laboratory and radiology investigations are seen as obstacles for an efficient flow since it is difficult to get fast results. The ED staff is pushing the radiology and laboratory staff with reminders that they are waiting for results. In reality the ED staff has minimal ability to speed up the workflow of the laboratory and radiology departments. The only thing they can influence is the speed of the delivery of blood samples to the laboratory.

The staff also acts with *long-term solutions* for speeding up the care. They push in order to change the laboratory and radiology work processes, and they push to get their own analytic equipment for blood tests since this would improve the flow. Issues and suggestions are discussed directly with managers and at work place meetings. The aim is to solve problems causing bad flow such as changing work procedures, improving collaboration with other departments, and extending laboratory services at the ED. A successful long-term structure pushing solution that bypass the waiting room was developed in ED3. This was done after the implementation of Lean production strategies [3] and was achieved by having a senior physician doing the first triage.

#### Shuffling around patients

To increase patient flow the staff needs to reprioritize the needs of patients waiting. They are then shuffling around patients, which is a frequent activity at ED1. It means that one patient has to leave the treatment room or the stand by waiting mode for a patient with a worse health status who needs a faster assessment. There are two main reasons for shuffling around patients.

First, when the number of treatment rooms is too small in relation to the number of patients. The staff then tries to juggle the shuffling of patients by *being one step ahead* and plan for the unforeseen. Many patients are moved to the stand by waiting mode after being examined in a treatment room so the next patient can be examined and then being moved back to the treatment room again.

Second, when the condition of a patient has deteriorated the situation may call for a shuffling of a patient into a treatment room, since a changed health condition requires immediate action. When the staff notices a critical situation approaching they act fast. A patient who is in a treatment room is moved out and a patient in stand-by mode who is deteriorating is moved in. The staff is *“zigzagging”* patients in and out of the treatment rooms so that they can treat those most in need of immediate treatment.

### Changing the waiting experience

To manage non-acceptable waiting the staff tries to alter the patients’ experience of waiting to make them feel it’s acceptable. This is done by *calming and informing* or by *feinting to cover up*.

#### Calming and informing

This is an important strategy at the ED and means that the staff tries to make the patient experience waiting as acceptable and the care as trustworthy and professional. Also, the waiting should not increase the patients’ worries about their state of health. The patients should feel safe and reassured that it will soon be their turn to see the physician. For the patient to feel calm the staff must be calm too. It is important for the staff to show respect and concern, and to take the time to listen to what the patients have to say since this promotes safety. To calm patients it is also important to inform them how the ED works, i.e. that those with the worst health status are always examined first. The staff knows that patients who are calmed and given this information will show less worries, irritation and fear, no matter where they are waiting. And the calming of patients has a soothing effect also on the accompanying persons’ stress and worries.

Another aspect of *informing patients* is to create comfort so that patients perceive the work at the ED as running normally.

#### Feinting to cover up

Feinting illusion maneuvers means one or several actions done in order to cover up and divert interest from a situation in order to change how it is perceived. The staff performs different feint maneuvers so the patient and accompanying person will experience waiting as acceptable. It is important to create this experience even if the staff considers the waiting non-acceptable. The goal is to achieve a calm and safe situation for patients and staff.

Feints are used to prevent the staff from having to answer questions they cannot answer such as “when can I come in?” and “when is the doctor coming?” Not knowing the answer to these questions is stressful for the staff. They want to know, but since they don’t know they avoid contact with patients in these situations. It is particularly stressful to see the disappointed face of a patient when the nurse looks into the treatment room instead of the long-awaited physician.

There are two types of feint maneuvers. In one type the staff is being visible and looking busy. The other type is when the staff make themselves invisible to make the patients believe that they are busy tending to patients elsewhere.

*Looking busy* can be used in waiting rooms, the stand-by mode and in treatment rooms. One example is when receptionists look only into their papers and avoid the patients’ glances. Another feint is when the nurse calls out the next in turn not looking into the waiting room. She then watches in her papers and turns around and looks only at the next patient in turn. To walk with determined steps and with a straight and busy glaze is another feint done in order not to be caught by the insistent looks from patients or accompanying persons who want attention. Carrying medical equipment or papers reinforces the illusion of being busy.

*Making oneself invisible* is expressed in different ways and at different locations such as hiding in the rinsing room or in the offices. The aim of making oneself invisible is to avoid confrontation with patients. Invisibility makes the staff inaccessible and they don’t risk getting caught by someone’s glance or word. “Invisibility striving” is a sign that the staff is busy and that waiting is necessary.

A common goal for the two types of feinting is to make patients and accompanying persons perceive waiting as necessary, and therefore it is important that the feinting is not discovered. And this may be the case if feinting maneuvers are used too much. Feint maneuvers are also risky since the staff may lose the necessary overview of patients, especially when they make themselves invisible. Staff that is hiding may also make patients and accompanying persons worried.

### Staff reactions to unsuccessful managing of non-acceptable waiting

The staff wants the patients and accompanying persons to experience good quality and high efficiency at the ED. And they don’t want them to perceive waiting as non-acceptable. The discrepancy between the care quality the staff wants to achieve and the actual quality of the care creates frustration of different types and the staff becomes upset and ashamed^a^. Frustration emerges when too many patients are waiting and when none of the previously mentioned strategies work to control the situation, and when non-acceptable waiting has not been reduced as much as expected.

So what happens when staff frustration gets out of hand since the waiting is non-acceptable and there are no more tools to manage it with? Shame then spreads and is expressed in different ways. The staff can feel ashamed in front of patients and accompanying persons when waiting is non-acceptable, or when the examination equipment is malfunctioning or missing. Shame is experienced as very demanding and everyone is doing what they can to cover their own shortcomings from the eyes of patients or accompanying persons.

When experiencing shame from shortcomings the staff acts in different ways. Either they avoid confronting patients or at least avoid meeting their eyes. Or they hide behind a shield of aggressiveness such as when they are angry at the slow pace of physicians, impatient family members or bad managers. Or when they consider someone arriving at the ED a wrong patient with no need for emergency care. These patients, of this they are convinced, should by definition be cared for by someone else, such as their family physician, which would make the ED less overcrowded.

In order to handle anger it happens that the staff let off pressure. In focus groups it was described as letting off steam or hiss which can be done in privacy or together with other staff members where the patient can’t hear. Letting off steam works as a vent for stressful job situations. It makes it easier to cope for some time. The positive side is that it ties at least some of the staff members closer together. The negative side is that accusations may evoke cross-pressures between the different professional groups. One group may feel exploited, having to work too hard etc.

Shame and anger can also be soothed by killing time. When the staff has tried all strategies to prevent a standstill at the ED and nothing happens, they engage in different activities to kill time. It can be gazing at the computer or reading a magazine, or fiddling with one’s cell phone. The last thing the staff wants is to appear idle.

The culmination of shame and anger may either result in protest or resignation. Protesting is an attempt to create attention for another structure push to try to change things to the better. Protesting can be done differently. At one occasion when the situation was very pressing a protest list was sent to both the media and the employer at the same time. The protest list theme explained consequences of bad working conditions at the ED. By protesting the staff wanted media coverage in order to push for improvements.

Engaging in dialoguing to achieve changes has a bad success record. One example of this is an attempt to get a new information sign in the waiting room. Despite complaints from both staff and patients that the text on the sign was wrong no new sign arrived. This caused distrust and the staff eventually protested but to no avail.

Resignation means that no one is expecting anything to happen. A bad work situation or unnoticed improvement ideas from the staff may lead to resignation. Resignation can manifest itself in different ways. Giving up, no more letting off steam, going on sick leave, or eventually quitting the job.

## Discussion

We did a classic grounded theory study by observing and interviewing with the aim to understand what happens at an ED when the waiting of patients is in focus. We found management of non-acceptable waiting as being central to the ED staff making a distinction between acceptable and non-acceptable waiting. The staff reacts in different ways when non-acceptable waiting cannot be reduced. They either explain away issues causing the waiting with “wrong patients” arriving at the ED, killing time through distractions, or eventually resigning from their jobs. Or, they successfully engage in waiting management, which means that actions are taken to either reduce the actual waiting or to change the waiting experience. Patient throughput is increased by structure pushing and shuffling around patients, and the actual waiting is reduced. Structure pushing involves strategies aimed at preventing or resolving problems or creating long term solutions. Calming, and feinting to cover up by illusionary maneuvers alter the patients’ experience of waiting. The staff finds it easier to reduce the perceived waiting than actual waiting [22], since it increases the degree of patient satisfaction [23].

Managing non-acceptable waiting at the ED is necessary for at least two reasons. First, it is essential for medical emergencies requiring immediate care. Not only qualitative data from ED1 supports this proposition but L.B and M-L.E also collected quantitative data from two other EDs. ED3 had a significant 20% lower 7-day mortality compared to ED1 and ED2 in a comparative analysis of 147.579 patients [3]. ED1 waiting management follows a traditional model with a registered nurse seeing the patient first. At ED2 a registered nurse does the first triage as well. At ED3 a senior physician sees the patient first which means that the ED3 waiting management has bypassed the waiting room with patients waiting in stand-by mode and investigation rooms only and thus reducing the waiting time by eliminating its first structure. The ED3 waiting management resembles an ED “process redesign” to increase throughput time, reduce waiting and increase patient satisfaction by a team based intervention that changed the ED work from top management to staff [24]. At another ED reorganization with an increment of the first physician triage competence was associated with a higher patient flow [25].

The second reason for minimizing non-acceptable waiting is that it is a social necessity since waiting is experiential and can be affected by the actions of the staff. The waiting experience may lead to anxiety, worries [26,27], anger and wrath [13,14]. The concepts of acceptable and non-acceptable waiting, the measures taken by the staff to manage this waiting, and the social necessity of reducing the waiting experience, raise the question of what waiting actually is. Dictionaries explain waiting in words such as “be patient”, “calm down”, “show patience”, “let the time pass”, “relate to”, “sit”, “look forward to”. The American sociologist Robbins was convinced that it is possible to affect a person’s waiting experience in different ways and with different measures since waiting basically is a neutral experience [28]. But, depending on the situation it can either be negative or positive. So, the waiting situation and the waiting circumstances will eventually determine a person’s reactions to waiting [28]. In a grounded theory study of patients waiting for a diagnosis the importance of information was evident: “seeking and giving information and interpreting clues moved the participants forward” [29]. In another grounded theory of patients and relatives in palliative care the main concern emerged as “living a life on hold”, a synonym to waiting. This led to insecurity and powerlessness, but the resolution was eventually to Decipher unwritten rules [30] requiring a lot of information to “Figure out” the rules in order to understand and deal with the waiting. In our study we found that the staff acts in order to reduce waiting and, as seen in other research, reduce the negative effects of overcrowding which is common in EDs all over the world. The staff’s definition of overcrowding is when there is an extensive waiting from ED arrival until patients meet the staff [31] Overcrowding often leads to lacking care that threatens patient safety [32]. As previously shown the staff in our study emphasized both the patients’ problems caused by overcrowding and the staffs’ own reactions to unsuccessful reduction of that waiting [14,32].

An unfamiliar and unknown environment with many ill people in combination with concerns about their own condition make patients at the ED worried and anxious. Therefore staff should care for patients as soon as they enter the waiting room. The patients need the presence of the staff, but also information since well-informed patients are calmer. This is part of a “process control” at the ED [33], which increases patient satisfaction even more if patients are informed about the expected waiting time [32].

The properties of waiting management that involve feinting and covering up to change the experience of waiting seem not so well studied systematically. In one study “Covering up for the doctor” was used in physician consultations by interpreters to avoid that threatening information was experienced as coming from the interpreters [34]. In another study patients were flattering the physicians in order to get what they wanted without waiting [35].

### Limitations

Most of the participant observation data from this study was done at one emergency department only - ED1. The generalizability of our proposed waiting management theory will thus have to be explored by future research. Yet, since a well done grounded theory should be abstract of time, place and people, we hope that readers of this paper recognize the transcendence of its central concepts.

## Conclusion

To manage non-acceptable waiting was a driving force behind much of the staff behavior at a Swedish ED. Increasing throughput of patient flow and changing the experience of waiting were the two main ways of waiting management that emerged from this study.

## Endnotes

^a^We agree with the definition of shame proposed by Scheffe (1990). He defines shame broadly as a name for a large family of emotions and feelings that arise through seeing self negatively, if even only slightly negatively, through the eyes of others, or only anticipating such a reaction.

## Competing interests

None of the authors report any competing interests.

## Authors’ contributions

LB, BS, M-LE did conceive of the study, collected data, analysed and interpreted data, and drafted the manuscript. HT analysed and interpreted data, and drafted the manuscript. All authors read and approved the final manuscript.

## Pre-publication history

The pre-publication history for this paper can be accessed here:

http://www.biomedcentral.com/1472-6963/13/95/prepub
